# Ultraviolet B Treatment of the Forearm Alters Supraspinal Nociceptive Processing

**DOI:** 10.1155/prm/6601529

**Published:** 2025-07-16

**Authors:** Peter D. Drummond, Lechi Vo, Matthew Carabetta

**Affiliations:** School of Psychology and Centre for Healthy Ageing, College of Health and Education, Murdoch University, 90 South Street, Murdoch, Western Australia 6150, Australia

**Keywords:** acoustic startle stimulus, hyperalgesia, inflammation, trigeminal nociceptive blink reflex, ultraviolet B radiation

## Abstract

Exposing the skin to high levels of ultraviolet B (UVB) radiation induces an inflammatory response that upregulates local nociceptive processing; this, in turn, facilitates protective responses to limit further injury. In this study, the UVB model was used to explore additional effects of inflammation on supraspinal nociceptive processing. Thirty-one healthy participants attended two sessions approximately 24 h apart. In each session, pressure-pain thresholds and sensitivity to sharp stimulation and heat were assessed in both forearms, and pressure-pain thresholds and sensitivity to sharp stimulation were assessed on each side of the forehead. In a novel paradigm, supraspinal nociceptive processing was explored by assessing pain and blink reflexes to electrical stimulation of the forehead, paired with acoustic startle stimuli. At the end of the first session, UVB radiation at a dose sufficient to induce erythema at the most exposed site was administered to one forearm. Consistent with local sensitization, sensitivity to heat and sharp stimulation had increased at the maximally exposed site 24 h later. This local response was accompanied by changes in supraspinal nociceptive processing—pressure-pain thresholds were lower on the ipsilateral than contralateral side of the forehead, and acoustic startle stimuli augmented electrically evoked pain. Blink reflexes weakened from the first to the second session, but decreases were smaller on the UVB-treated than contralateral side. Together, these findings suggest that acoustic startle stimuli facilitated activity in sensitized supraspinal nociceptive pathways. Potentially, this supraspinal mechanism adds to the burden of chronic nociplastic pain during states of heightened arousal and stress.

## 1. Introduction

Acute inflammation triggers neural sensitization both at the site of inflammation and within the central nervous system [1]. This sensitization usually resolves as inflammation subsides. However, persistence of sensitization may facilitate the transition from acute to chronic pain [2, 3], ultimately resulting in widespread hyperalgesia and other nociplastic symptoms associated with chronic pain [4].

Animal and human models of inflammation have been developed to clarify the steps involved in this transition. One such human model involves exposing the skin to high levels of ultraviolet B (UVB) radiation [5–7]. In this model, inflammatory mediators such as tumour necrosis factor *α* and interleukin 6 [8] sensitize peripheral nociceptors in the inflamed skin which, in turn, increases sensitivity to heat and mechanical stimuli [9–11]. Under some conditions, mechanical sensitivity extends into surrounding skin [12], consistent with a facilitatory influence on activity in spinal nociceptive projection neurons [11].

UVB treatment may also provoke changes in supraspinal nociceptive processing. In our previous work, UVB treatment of the forearm triggered a decrease in pressure-pain thresholds in the ipsilateral forehead [13], akin to the hemilateral hyperalgesia associated with complex regional pain syndrome (CRPS) [14]. Pain evoked by electrical stimulation of the forehead decreased when a control site was heated, consistent with conditioned pain modulation. However, pain remained stable when the UVB-treated site was heated, suggesting that an ipsilateral facilitatory mechanism masked inhibitory influences on pain.

The aim of the present study was to explore further characteristics of this putative supraspinal pronociceptive mechanism. Sudden loud acoustic stimuli initiate an arousal/alarm response and defensive behaviours that help to protect against severe injury during surprise attacks [15, 16]. This startle response may draw attention to noxious sensations in vulnerable parts of the body. As sensitization serves a similar purpose, we hypothesized that an acoustic startle stimulus would intensify painful sensations in the ipsilateral forehead after the UVB treatment and facilitate nociceptive components of the electrically evoked blink reflex [17]. In addition, we expected that the supraspinal pro-nociceptive mechanism would augment auditory discomfort and defensive reflexes—in the present case, the blink reflex to the acoustic startle probe.

Although mechanical sensitivity after UVB exposure typically is limited to irradiated skin [7, 13], mechanical sensitivity may extend into surrounding skin after more extensive exposure to UVB [12, 18]. In our previous work, UVB treatment triggered changes in supraspinal nociceptive processing in the absence of mechanical hyperalgesia around the exposed site [13]. However, this finding was novel and has yet to be confirmed. Thus, an additional aim of this study was to verify that UVB radiation at a dose that would not be expected to evoke signs of secondary hyperalgesia would nevertheless alter supraspinal nociceptive processing.

## 2. Methods

### 2.1. Participants

The sample consisted of 15 males and 16 females aged between 18 and 52 years (mean age ± standard deviation 26.5 ± 7.7 years) who participated in the study for course credit or who were recruited via personal contacts. Exclusion criteria included pregnancy or breastfeeding, chronic pain, medical treatment for a psychiatric disorder, consumption of analgesic medication, and heightened vulnerability to sunburn (e.g., via consumption of medications that increase sensitivity to UVB radiation). Participants provided their informed written consent for the procedures, which were approved by the Murdoch University Human Research Ethics Committee. Members of the public appointed to this committee provided feedback on draft applications and approved the final ethics submission.

### 2.2. Overview of the Experimental Design and Power Size Calculation

**Figure 1:**
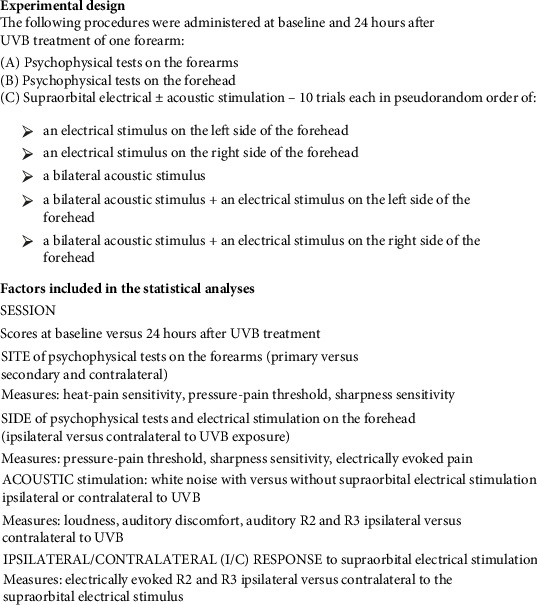
Summary of the experimental design and factors included in statistical analyses. Factor names are listed in upper case and levels within each factor are described in lower case. For SITE, the primary site refers to the site of maximal exposure to UVB whereas the secondary site refers to an unexposed site 2–3 cm away from UVB exposure.

Participants attended two sessions separated by approximately 24 h in a temperature-controlled laboratory. During each session, pressure-pain thresholds and sensitivity to sharp stimulation and heat were assessed in both forearms, and pressure-pain thresholds and sharpness were assessed in the forehead (Figure 1). In addition, blink reflexes to the following stimuli were recorded: an electrical stimulus on either the left or right side of the forehead; a bilateral acoustic stimulus; and a bilateral acoustic stimulus + an electrical stimulus on either the left or right side of the forehead. Ten trials of each stimulus type were administered at 20–30 s intervals in the same pseudorandom order to each participant with the provision that no more than two electrical stimuli were delivered sequentially on the same side.

At the end of the first session, one of the participant's forearms was exposed to UVB radiation. Twenty-four hours later, when inflammation had developed, the psychophysical and blink reflex procedures were repeated.

G^∗^Power analysis [19] indicated that a sample size of 26 was required to detect a strong effect (partial eta squared [*η*^2^] = 0.08) for the 2 × 2 × 2 repeated measures interaction (SESSIONS [before UVB, after UVB] x SIDE [ipsilateral, contralateral] x ACOUSTIC [yes, no] or SITE [primary, secondary]) with power of 0.80, *α* of 0.05 and option “as in SPSS”. The final sample of 31 was larger than samples in similar studies [13, 20–22] and thus was expected to be sufficient to identify moderate-to-strong effects.

### 2.3. Psychophysical Assessment

Procedures were similar to those reported previously [20]. In brief, participants initially were trained in the procedures until ratings and pressure-pain thresholds stabilized. Sensitivity to sharp (“pinprick”) sensations was assessed on the volar aspect of both forearms approximately 3 and 6 cm below the antecubital crease, followed by pressure-pain thresholds and sensitivity to heat. Pressure-pain thresholds and sensitivity to pinprick were then assessed bilaterally 2-3 cm above the supraorbital ridge. The side tested first alternated between one participant and the next. To minimise carry-over effects, tests at each site were separated by a few minutes.

To assess sensitivity to pinprick sensations, a nylon monofilament with a bending force of 10 g (Neuro-pen, Owen Mumford, USA) was applied for 2 s. Participants rated sharpness and pain on 0–10 verbal rating scales where 0 corresponded to “not sharp” or “not painful”, 2–3 to “slightly”, 5 to “moderately”, 7–8 to “between moderately and extremely” and 10 to “extremely sharp” or “extremely painful”. However, as pain was minimal, only ratings of sharpness are reported. To measure pressure-pain thresholds, an algometer (FDX, Wagner Instruments, USA) fitted with an 8 mm diameter hemispheric rubber tip on the plunger (to avoid edge effects) was applied at 100 g/sec until the participant reported pain. To assess sensitivity to heat, radiant heat from a purpose-built servo-controlled lamp was focused onto a 3 mm diameter patch of skin. The skin was heated to 44 ± 0.2°C within 1 s and maintained at that temperature for a further 6 s. Skin temperature was monitored continuously with a thermistor that controlled the intensity of radiant heat throughout the 7 s stimulation period. After the heating period, heat-pain was rated verbally on the 0–10 verbal pain rating scale described above. In our laboratory, test-retest reliability is good for pressure-pain thresholds and moderate for ratings of heat-pain and pinprick sensations [23, 24].

### 2.4. Blink Reflexes

Two custom-built concentric electrodes, each consisting of a thin copper wire cathode centered within a ring-shaped stainless-steel anode with an inner diameter of 10 mm and a contact outer diameter of 20 mm, were smeared with conductive gel and attached to the supraorbital region on each side of the forehead with adhesive tape. To elicit blink reflexes, a 2-mA monopolar square-wave triple-pulse train with 0.5 ms pulse duration and an inter-pulse interval of 5 ms was delivered via the cathode of one of the concentric electrodes from a DS7A Digitimer (Welwyn Garden City, UK). Triple-pulse electrical stimulation increases pain and facilitates R2, and therefore is more suitable for examining nociceptive processes than single pulses [25]. After each electrical stimulus, participants rated pain verbally on the 0-10 scale. To evoke an acoustic blink reflex, a 50 ms burst of white noise at 110 dB SPL was administered binaurally via 3M E-A-RTONE Gold 3A audiometric insert earphones (3M Auditory Systems Inc., USA) fitted with disposable foam ear inserts. For dual stimuli, the acoustic and electrical stimuli began simultaneously, and the acoustic stimulus continued for 38.5 ms after the offset of the triple-pulse train. After each acoustic stimulus, participants rated loudness and auditory discomfort verbally on 0-10 scales.

To detect blink reflexes, modified disposable Cleartrode electrodes (ConMed Corporation, NY, USA) were attached over the orbicularis oculi muscle of the lower eyelid and the outer corner of each eye. A ground electrode was attached behind the right ear. Biopotentials were amplified with an electromyograph amplifier (Biopac Systems, Inc., USA), digitized by an MP100 Biopac Systems Analogue/Digital Channel receptor at 2000 Hz and displayed on a computer monitor using AcqKnowledge software (Biopac Systems). Where appropriate, frequencies below 10 Hz and electrical noise at 50 Hz were filtered. The area under the curve of the rectified signal was measured for the second (R2) component of the blink reflex between 27 and 87 ms after stimulus onset and for the third component (R3) between 70 and 130 ms after stimulus onset [26]. Electromyographic activity was also recorded during a 10 s maximum voluntary contraction of the orbicularis oculi muscles. The blink reflex was not assessed in one participant with acute strabismus.

### 2.5. UVB Treatment

At the end of the first session, the volar aspect of the left or right forearm (counterbalanced across participants) was exposed to UVB radiation from a lamp with an irradiance energy level of 6 mW/cm^2^ (Durham Erythema Tester Device; Hybec, UK) [27]. The lamp was positioned to avoid irradiating tattoos or other significant skin features such as scars or moles, and was fitted with a graduated filter across 10 1-cm diameter sites such that radiation was attenuated 0% close to the antecubital crease (where pressure-pain thresholds and sensitivity to pinprick and heat-pain had been assessed; referred to below as the site of primary hyperalgesia) down to 84% at the most filtered site. The second more distal site of psychophysical assessment in the forearm (referred to below as the site of secondary hyperalgesia) was 2–3 cm away from sites of UVB exposure. As we aimed to induce only minor skin inflammation, the duration of exposure was adjusted for skin pigmentation in line with calibration charts provided with the lamp that listed the duration required to induce erythema for different skin types [28] together with the attenuation of UVB exposure from one filtered site to the next. Thus, the duration of exposure ranged from 65 s (0.39 mW/cm^2^ at the most exposed site) for participants with pale white skin (Fitzpatrick skin type I) to 250 s (1.5 mW/cm^2^ at the most exposed site) for participants with dark brown skin (Fitzpatrick skin type V). Overall, the mean duration ± S.D. was 167 ± 60 s. None of the participants had Fitzpatrick Skin Type VI (deeply pigmented dark brown to black skin) [28].

### 2.6. Second Session

Twenty-four hours later, the skin exposed to UVB radiation was inspected visually for signs of local inflammation and the psychophysical and blink reflex procedures were repeated.

### 2.7. Statistical Approach

The focus of the analysis was on intraindividual change both across time and sites (i.e., each subject acted as their own control). Preliminary analyses with the Shapiro–Wilk test indicated that scores for some variables differed significantly from a bell-shaped distribution. However, hypotheses were investigated using analysis of variance for repeated measures as this procedure is robust to moderate violations of the normality assumption and permits investigation of interactions among repeated measures factors [29]. Factors included in the statistical analyses are described in Figure 1.

#### 2.7.1. Sensory Effects of UVB Exposure

Scores for the two forearm sites contralateral to UVB exposure were averaged. Changes in sensitivity in the forearms to mechanical and thermal stimuli 24 h after exposure to UVB radiation were investigated in SESSION (baseline, 24 h later) x SITE (primary, secondary, contralateral) repeated measures analyses of variance. Where necessary, the Greenhouse-Geisser epsilon was used to correct for violations of the sphericity assumption. In this and the following analyses, simple main effects that contributed to significant interactions were investigated within and across sites using paired *t*-tests (i.e., differences across levels of one factor were investigated within each level of the second factor). For factors with three levels, the criterion of statistical significance was adjusted using the Bonferroni procedure for multiple comparisons. No adjustment was made for factors with two levels as only one comparison between those levels was possible.

Changes in sensitivity in the forehead to mechanical stimuli 24 h after the UVB treatment were investigated in SESSION (baseline, 24 h later) x SIDE (stimulus administered ipsilateral versus contralateral to UVB exposure) repeated measures analyses of variance. Analysis of pain ratings to electrical stimulation of the forehead had an additional factor of ACOUSTIC (electrical stimulus alone versus combined electrical and acoustic stimuli).

Ratings of loudness and auditory discomfort to the bilateral acoustic stimulus were investigated in SESSION (baseline, 24 h later) x ACOUSTIC (acoustic stimulus alone, electrical stimulus ipsilateral to UVB exposure combined with an acoustic stimulus, electrical stimulus contralateral to UVB exposure combined with an acoustic stimulus) repeated measures analyses of variance.

#### 2.7.2. Effect of UVB Exposure on Blink Reflexes

Within each session, the R2 and R3 components of the blink reflex were averaged across the 10 trials of each stimulus type. Each component of the response to acoustic stimuli was investigated in a SESSION × SIDE repeated measures analysis of variance. Analyses of responses to electrical stimuli included additional factors of ACOUSTIC (electrical stimulus alone versus electrical stimulus combined with an acoustic stimulus) and I/C RESPONSE (blink reflex ipsilateral versus contralateral to the supraorbital electrical stimulus).

In all analyses, the criterion of statistical significance was *p* < 0.05.

## 3. Results

In all 31 cases, erythema was visible at the site of maximal exposure 24 h after the UVB treatment but none of the participants reported that the site was spontaneously painful. In some cases, erythema was also clearly visible at sites where the graduated filter attenuated UVB exposure to 90% or 75% of maximum.

### 3.1. Sensory Effects of UVB Exposure

#### 3.1.1. Forearm Sharpness Ratings

**Figure 2:**
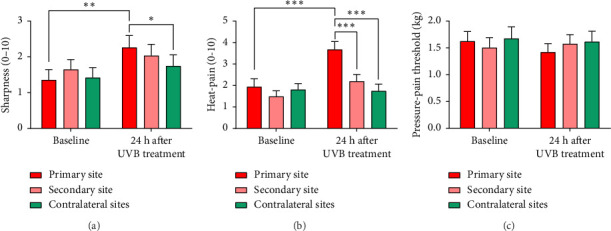
Sensitivity ± standard error in the forearms to (a) sharpness and (b) heat-pain, and (c) pressure-pain thresholds before and 24 h after one forearm was exposed to UVB radiation. Sensitivity was assessed at the site of maximum UVB exposure (red bars), 3 cm away in the same forearm (pink bars), and at mirror image sites in the contralateral forearm (dark green and light green bars). Twenty-four hours after the UVB treatment, sharpness and heat-pain were greater at the site of maximum UVB exposure than at baseline and were greater at this site than in the contralateral forearm (consistent with primary hyperalgesia to sharpness and heat). Similar effects were identified for sharpness 3 cm from the site of maximum UVB exposure (consistent with secondary hyperalgesia). ^∗^*p* < 0.05; ^∗∗^*p* < 0.01; ^∗∗∗^*p* < 0.001.

Relative to contralateral sites, sensitivity to sharp stimulation increased at the maximally exposed site, consistent with the development of primary hyperalgesia [SESSION × SITE interaction: *F* (1.53, 45.81) = 4.44, *p*=0.026, partial *η*^2^ = 0.129] (Figure 2(a)). A trend for ratings to be higher after the UVB treatment at the secondary site than at contralateral sites [*t* (30) = 2.47, *p*=0.020] did not survive Bonferroni correction. Whether this trend was due to UVB exposure is uncertain, as increases in sharpness from before to after the UVB treatment were similar at the secondary and contralateral sites.

#### 3.1.2. Forearm Heat Ratings

Primary hyperalgesia to heat developed at the maximally exposed site [SESSION × SITE interaction: *F* (1.62, 48.57) = 8.71, *p*=0.001, partial *η*^2^ = 0.225] (Figure 2(b)). Sensitivity to heat was slightly greater at the secondary site after the UVB treatment than at baseline [*t* (30) = 2.20, *p*=0.035] but did not differ from sensitivity at contralateral sites.

#### 3.1.3. Forearm Pressure-Pain Thresholds

Pressure-pain thresholds remained largely unchanged after the UVB treatment [SESSION × SITE interaction: *F* (2, 60) = 3.08, *p*=0.053, partial *η*^2^ = 0.093] (Figure 2(c)), apart from a slight decrease below baseline in the pressure-pain threshold at the primary site [*t* (30) = 2.18, *p*=0.037].

#### 3.1.4. Supraspinal Mechanical Hyperalgesia

**Figure 3:**
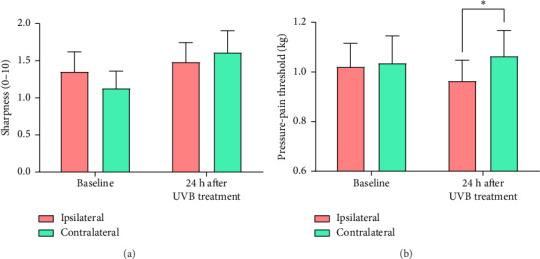
Measure ± standard error on each side of the forehead for (a) sharpness and (b) pressure-pain thresholds before and 24 h after one forearm was exposed to UVB radiation. The red bars represent sensitivity ipsilateral to the UVB treatment and the green bars represent sensitivity contralateral to the UVB treatment. ^∗^*p* < 0.05.

Sensitivity in the forehead to sharp stimulation remained stable after the UVB treatment (Figure 3(a)) whereas pressure-pain thresholds diverged at the treated and untreated sides [SESSION × SIDE interaction: *F* (1, 30) = 5.63, *p*=0.024, partial *η*^2^ = 0.158] (Figure 3(b)). Investigation of this interaction indicated that pressure-pain thresholds were significantly lower on the ipsilateral than contralateral side of the forehead 24 h after the UVB treatment (Figure 3(b)).

#### 3.1.5. Electrically Evoked Pain

**Figure 4:**
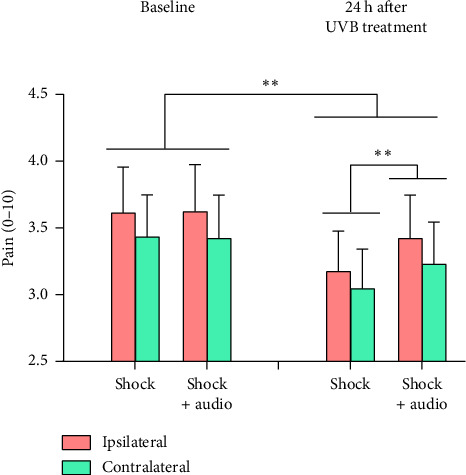
Pain ratings ± standard error to supraorbital electrical stimulation before and 24 h after one forearm was exposed to UVB radiation. The red bars represent sensitivity ipsilateral to the UVB treatment and the green bars represent sensitivity contralateral to the UVB treatment. ^∗∗^*p* < 0.01.

Pain ratings to electrical stimulation of the forehead were higher before than 24 h after the UVB treatment [main effect for SESSION, *F* (1, 30) = 9.31, *p*=0.005, partial *η*^2^ = 0.237] but concurrent acoustic stimulation moderated this effect [SESSION × ACOUSTIC interaction, *F* (1, 30) = 9.42, *p*=0.005, partial *η*^2^ = 0.239] (Figure 4). Investigation of the simple main effects that contributed to this interaction indicated that pain ratings to the electrical stimulus were higher in Session 1 than Session 2 both with and without concurrent acoustic stimulation (in line with the significant main effect for SESSION reported above). The acoustic stimulus did not affect pain ratings at baseline; however, 24 h after exposure to UVB, pain ratings were higher to combined acoustic and electrical stimuli than to the electrical stimulus alone (*p* < 0.01) (Figure 4).

#### 3.1.6. Auditory Sensitivity

**Figure 5:**
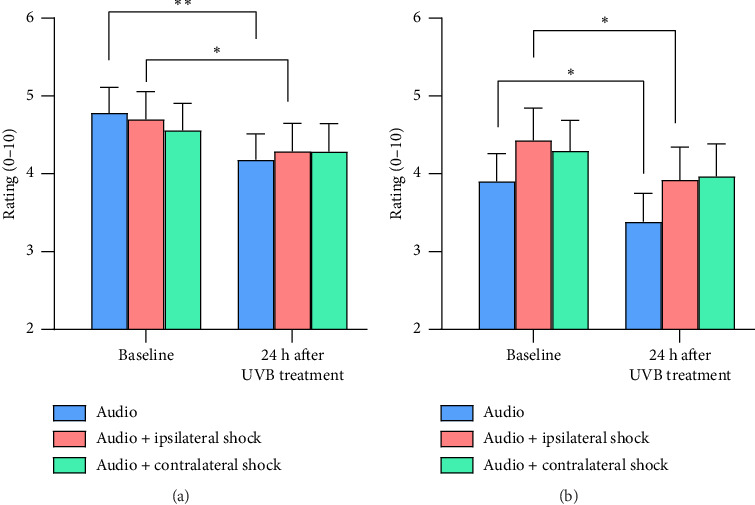
Ratings ± standard error of (a) loudness and (b) auditory discomfort to acoustic stimuli presented alone (blue bars) or together with supraorbital electrical stimulation ipsilateral (red bars) or contralateral (green bars) to the UVB treatment. ^∗^*p* < 0.05; ^∗∗^*p* < 0.01.

Ratings of loudness and auditory discomfort declined from before to after the UVB treatment, but this depended on whether the acoustic stimulus was delivered alone or together with supraorbital electrical stimulation [SESSION × ACOUSTIC interaction: for loudness *F* (2, 29) = 5.16, *p*=0.012, partial *η*^2^ = 0.262; for auditory discomfort *F* (2, 29) = 3.58, *p*=0.041, partial *η*^2^ = 0.198]. Investigation of the simple main effects that contributed to these interactions indicated that loudness (*p* < 0.01, Figure 5(a)) and auditory discomfort (*p* < 0.05, Figure 5(b)) were lower 24 h after the UVB treatment than at baseline when the acoustic stimulus was delivered alone. In addition, loudness (*p* < 0.05, Figure 5(a)) and auditory discomfort (*p* < 0.05, Figure 5(b)) were lower 24 h after the UVB treatment than at baseline when the acoustic stimulus was paired with electrical stimulation of the ipsilateral forehead but remained stable when the acoustic stimulus was paired with electrical stimulation of the contralateral forehead.

### 3.2. Effects of UVB Exposure on Blink Reflexes

To control for effects of electrode placement and impedance on electromyographic activity, The R2 and R3 components of the blink reflex were expressed as the proportion of maximum voluntary contraction of the orbicularis oculi muscles in the results presented below. Results were similar for raw values (Supporting Tables e.7 and e.8). Electrical stimuli did not evoke a short-latency ipsilateral R1.

#### 3.2.1. Acoustic Stimulus

**Figure 6:**
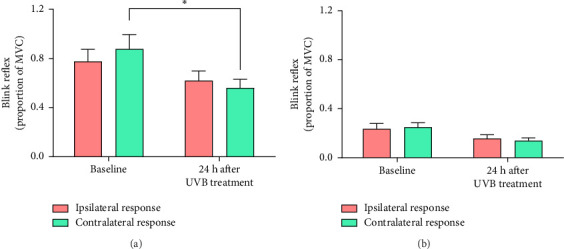
(a) R2 and (b) R3 ± standard error to acoustic stimuli before and 24 h after one forearm was exposed to UVB radiation. The red bars represent blink reflexes ipsilateral to the UVB treatment and the green bars represent blink reflexes contralateral to the UVB treatment. R2 and R3 are expressed as a proportion of maximum voluntary contraction (MVC) of the orbicularis oculi muscles. ^∗^*p* < 0.05.

The R2 component of the blink to the acoustic stimulus was lower than baseline 24 h after the UVB treatment [*F* (1, 29) = 5.18, *p*=0.030, partial *η*^2^ = 0.152], but this was moderated by the UVB treatment [SESSION × SIDE interaction, *F* (1, 29) = 5.74, *p*=0.023, partial *η*^2^ = 0.165] (Figure 6(a)). Investigation of the simple main effects that contributed to this interaction indicated that R2 contralateral to the UVB treatment was lower than baseline 24 h after the UVB treatment (*p* < 0.05) whereas R2 ipsilateral to the UVB treatment remained stable (Figure 6(a)). The R3 component was minimal in both sessions (Figure 6(b)), suggesting that the acoustic stimulus evoked an acoustic blink reflex rather than a broader long-latency startle response [30].

#### 3.2.2. Electrically Evoked Blink Reflex

**Figure 7:**
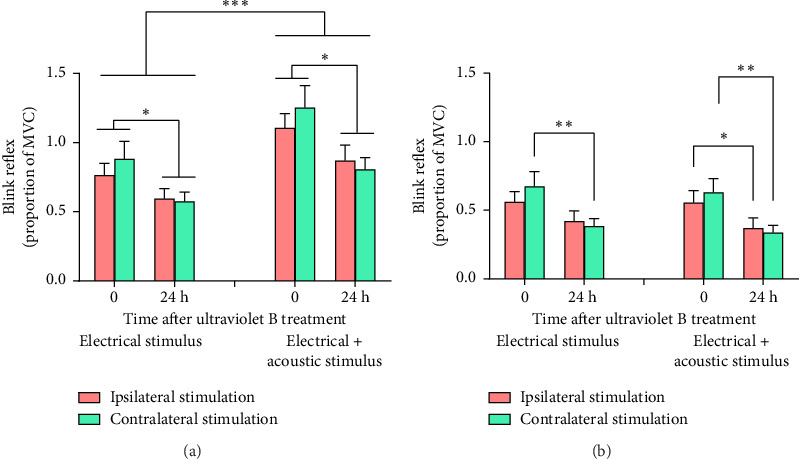
(a) R2 and (b) R3 ± standard error to blinks (averaged across sides) evoked by electrical stimulation of the forehead ipsilateral (red bars) or contralateral (green bars) to the side of UVB treatment. R2 and R3 are expressed as a proportion of maximum voluntary contraction (MVC) of the orbicularis oculi muscles. ^∗^*p* < 0.05; ^∗∗^*p* < 0.01; ^∗∗∗^*p* < 0.001.

Overall, R2 was greater ipsilateral than contralateral to the supraorbital electrical stimulus [0.90 ± 0.079 normalised units versus 0.81 ± 0.077 normalised units, main effect for I/C RESPONSE, *F* (1, 29) = 79.8, *p* < 0.001, partial *η*^2^ = 0.734]. The lateralised response persisted across sessions and was unaffected by the UVB treatment. Similarly, R3 was greater ipsilateral than contralateral to supraorbital electrical stimulation [0.53 ± 0.065 normalised units versus 0.46 ± 0.059 normalised units, main effect for I/C RESPONSE, *F* (1, 29) = 47.3, *p* < 0.001, partial *η*^2^ = 0.620], and this asymmetry was unaffected by the UVB treatment. Therefore, the blink to electrical stimulation was averaged across sides (illustrated in Figure 7).

The acoustic stimulus facilitated electrically evoked R2 in both sessions [main effect for ACOUSTIC, *F*(1, 29) = 82.5, *p* < 0.001, partial *η*^2^ = 0.740] (Figure 7(a)). R2 declined after the UVB treatment [main effect for SESSIONS, *F* (1, 29) = 6.58, *p*=0.016, partial *η*^2^ = 0.158] (Figure 7(a)); a trend for the decrease to be smaller for electrical stimuli delivered ipsilateral than contralateral to the treated arm did not achieve statistical significance [SESSION × SIDE interaction: *F* (1, 29) = 3.82, *p*=0.060, partial *η*^2^ = 0.116].

In contrast to R2, the acoustic stimulus did not facilitate R3. R3 was lower than baseline 24 h after exposure to UVB radiation [main effect for SESSION, *F*(1, 29) = 7.58, *p*=0.010, partial *η*^2^ = 0.207] (Figure 7(b)) but the UVB treatment moderated this decrease [SESSION × SIDE interaction: *F*(1, 29) = 5.49, *p*=0.028, partial *η*^2^ = 0.159] (Figure 7(b)). Investigation of the simple main effects that contributed to this interaction indicated that R3 to electrical stimulation delivered contralateral to the treated arm (alone and with concurrent acoustic stimulation) was smaller 24 h after the UVB treatment than at baseline (*p* < 0.01) whereas the decrease in R3 to electrical stimulation delivered ipsilateral to the treated arm (alone and with concurrent acoustic stimulation) was more variable (*p*=0.050). Further exploration of these effects indicated that R3 to electrical stimulation delivered ipsilateral to the treated arm was smaller 24 h after the UVB treatment than at baseline when paired with acoustic stimulation (*p* < 0.05) but not when administered alone (Figure 7(b)).

## 4. Discussion

The aim of this study was to investigate supraspinal nociceptive processing in the UVB model of inflammation. Startle stimuli and hyperalgesia each evoke defensive behaviours to limit harm. Hence, after the UVB treatment, we hypothesized that acoustic startle stimuli would augment pain and blink reflexes to electrical stimulation of the forehead; and, conversely, that the UVB treatment would enhance auditory discomfort and blink reflexes to the acoustic startle stimulus.

Both hypotheses were supported, in part. After the UVB treatment, the acoustic startle stimulus augmented pain evoked by electrical stimulation of the forehead. In addition, electrically evoked R3 and auditory R2 declined more from the first to the second session contralateral than ipsilateral to the UVB treatment. Together, the findings suggest that acute inflammation in the UVB model altered supraspinal pain processing which, in turn, promoted interaction between the arousal/alarm and nociceptive systems.

### 4.1. Sensory Effects of UVB Exposure in the Treated Forearm

#### 4.1.1. Primary Hyperalgesia

UVB radiation damages skin cells; this initiates a local inflammatory response that sensitizes axons and nerve endings in the exposed skin [31] and triggers hyperalgesia that peaks over the next 48 h [6, 12]. We adjusted the UVB dose individually to induce only minor erythema in a small patch of skin. Nonetheless, 24 h later, the skin at the maximally exposed site was red and sensitive to heat and sharp stimulation. Thus, it seems likely that inflammatory mediators produced in response to the UVB treatment sensitized polymodal nociceptors in the exposed skin [32, 33].

#### 4.1.2. Secondary Hyperalgesia

UVB radiation does not normally evoke spontaneous pain [12] but, nevertheless, may trigger changes in spinal pain processing [11], presumably due to sporadic but protracted low-frequency nociceptive input [34]. In the present study, there was only subtle evidence of secondary hyperalgesia around the UVB-treated site, possibly because the UVB intensity and area of exposure were minimal (thus limiting temporal and spatial summation of nociceptive signals). Top-down modulation of nociceptive neurotransmission in the spinal dorsal horn has been investigated extensively in animal models of inflammation [2, 3, 35]. Apart from pain circuits with direct input from inflamed tissue, facilitatory influences that descend from the rostral ventrolateral medulla predominate over inhibitory influences, and hence augment secondary mechanical hyperalgesia around the site of inflammation [2]. This facilitatory influence likely originates in the mediodorsal thalamic nuclei and promotes secondary hyperalgesia to mechanical but not heat stimuli [36]. Whether secondary hyperalgesia is detected after UVB treatment [12, 18, 37, 38] [but see 7, 13] may depend on the extent of exposure or on ambient conditions because heating the treated site unmasks signs of central sensitization [11].

### 4.2. Sensory Effects in the Forehead to UVB Treatment of the Forearm

#### 4.2.1. Mechanical Hyperalgesia in the Ipsilateral Forehead

We reported previously that pressure-pain thresholds had decreased in the ipsilateral forehead 48 h after the forearm was exposed to twice the minimum erythema dose of UVB [13]. In the present study, pressure-pain thresholds had decreased in the ipsilateral forehead, relative to contralateral changes, 24 h after UVB treatment of the forearm at close to the minimum erythema dose. Together, these findings suggest that low-grade inflammation triggers an ipsilateral supraspinal facilitatory influence on pain processing. Under more extreme conditions, this supraspinal influence may also contribute to spinal sensitization [11, 12]. In contrast to pressure-pain thresholds, pinprick ratings remained stable, possibly because different neural mechanisms contribute to cutaneous sensitivity versus deep-pressure hyperalgesia [39].

#### 4.2.2. Perception of Electrical Stimuli

Pain ratings to electrical stimulation of the forehead decreased from the first to the second session, as did ratings of loudness and auditory discomfort to acoustic startle stimuli, probably as participants became more familiar with the procedures. However, after the UVB treatment, acoustic stimuli boosted electrically evoked pain, consistent with convergence of arousal/alarm signals on sensitized supraspinal nociceptive circuits (e.g., in the thalamus).

#### 4.2.3. Auditory Sensitivity

Contrary to expectations, electrical stimulation of the ipsilateral forehead did not enhance auditory discomfort after the UVB treatment. Nonetheless, the findings point to a specific effect of the UVB treatment on pain rather than a nonspecific bias toward all sensations on the treated side.

### 4.3. Effects of the UVB Treatment on Blink Reflexes

#### 4.3.1. Electrically Evoked Blink Reflexes

At low stimulus intensities, the high current density at the cathode of concentric electrodes preferentially depolarizes superficial nociceptive Aδ fibres [40]. Supraorbital electrical stimuli that activate nociceptive afferent Aδ-fibres in the trigeminal nerve trigger the bilateral R2 component of the electrically evoked blink reflex, and possibly also R3, whereas non-nociceptive fibres mediate the initial ipsilateral R1 wave [26, 41]. In addition, a startle reaction that habituates rapidly to repeated stimulation may contribute to the R3 component of the electrically evoked blink reflex [42]. The weak electrical stimuli delivered from concentric electrodes in the present study elicited R2 and R3 but not R1, consistent with preferential excitation of superficial nociceptors [22, 40]. Additionally, factors such as habituation may have influenced R2 and R3 as these responses weakened from the first to the second session.

Despite this, decreases in R3 (and a similar trend for R2) were smaller for supraorbital electrical stimuli delivered ipsilateral than contralateral to the UVB-treated arm. In our previous work, high-frequency electrical stimulation of the forearm evoked signs of spinal sensitization and facilitated ipsilateral R2 [21], suggesting that the facilitatory influence that drives spinal sensitization [2, 36] also projects to trigeminal nociceptive pathways in the brainstem. This facilitatory influence might explain why declines in the electrically evoked blink reflex were smaller on the UVB-treated side.

The acoustic startle stimulus facilitated the electrically evoked R2 [43], likely due to convergence of neurotransmission in the reticular formation or upon the motor neurons that propel the blink [15]. However, the UVB treatment did not enhance the response to the combined acoustic and electrical stimulus, possibly due to a ceiling effect as blink reflexes were close to maximum.

#### 4.3.2. Acoustic Blink Reflexes

Although mediated primarily by activity in the midbrain reticular formation [30, 44], psychological arousal and emotions such as fear enhance the blink to acoustic probes [45] whereas pleasant emotions suppress these blinks [46]. In the present study, the reflex ipsilateral to the UVB treatment decreased less across sessions than the contralateral reflex. The blink to an acoustic probe delivered to just one ear is greater on this than the contralateral side [46]. Therefore, we speculate that prior exposure to the UVB treatment weighted responses toward the treated side in the present study.

## 5. Limitations

For ethical reasons, the dose of UVB radiation was titrated in proportion to skin pigmentation. Although this helped to control for individual differences, effects might have been more pronounced with higher doses of UVB. Specifically, floor effects may have affected assessment of skin sensitivity because most pain ratings were low. However, we are confident that the lateralized changes identified in this study were due to the UVB treatment as each participant served as their own control. A shortcoming of this approach is that we did not control for effects of repeated testing. Thus, we do not know whether general changes in ratings and blink reflexes from the first to the second session were due to the UVB treatment, to habituation or to nonspecific decreases in arousal or anxiety.

UVB stimulation altered ipsilateral blink reflexes and pressure-pain thresholds in the ipsilateral forehead, relative to the contralateral side. However, effects of the UVB treatment on heat-pain in the ipsilateral forehead were not explored due to technical constraints. It would be interesting to investigate this in future studies as effects of UVB stimulation in the forearm were strongest for heat-pain. It would also be interesting to assess thresholds to other forms of stimulation (e.g., electrical) for direct comparison to changes in the pressure-pain threshold.

The UVB treatment appeared to influence both R2 and R3, but whether this was mediated by nociceptive or startle circuits is uncertain as R3 (and possibly R2) is affected by attention in addition to pain [26, 42, 47, 48]. In practice, these two components of the electrically evoked blink are sometimes difficult to tease apart due to substantial overlap. More specific forms of nociceptive stimulation (e.g., laser) [48] may be required to clarify effects of UVB treatment on the supraspinal modulation of pain.

Finally, the participants in this study were young, healthy males and females. Therefore, the findings might not generalise to people in other age groups or with health complaints or chronic pain.

## 6. Conclusions and Clinical Implications

Overall, the findings indicate that UVB-inflammation altered nociceptive activity not only at the exposed site but also within the central nervous system. We hypothesize that facilitatory influences stemming from the dorsomedial thalamus and rostral ventrolateral medulla that contribute to secondary hyperalgesia during acute inflammation [2, 36] promote mechanical hyperalgesia more widely. In the short term, hyperalgesia on the injured side of the body could heighten awareness of potential threats in the general vicinity of inflamed and vulnerable tissue, thereby fostering protective behaviours.

The present findings indicate that startle stimuli engage this protective response. We speculate that convergence of arousal/alarm signals on sensitized nociceptive circuits increases awareness of sensations in vulnerable parts of the body (e.g., near the eyes). This neural crosstalk may explain why startle stimuli provoke pain in conditions such as CRPS and why pain increases during psychological stress in CRPS and other forms of chronic pain [49–52]. Clarifying the mechanisms that underpin interactions between the arousal/alarm and nociceptive systems is imperative, as unchecked facilitatory influences on pain might ultimately result in widespread hyperalgesia and other nociplastic symptoms in patients with chronic pain [14, 49, 53–56].

## Data Availability

The data that support the findings of this study are available from the corresponding author upon reasonable request.

## Supporting Information

Descriptive and inferential statistics are presented in Supporting files: for psychophysical assessments (Tables e.1 and e.2); for pain ratings to electrical stimuli ipsilateral and contralateral to the treated arm, and loudness and auditory discomfort to acoustic stimuli (Table e.3 and Table e.4); for the R2 and R3 components of the blink reflex to the acoustic stimulus (Table e.5 and Table e.6); and for the R2 and R3 components of the blink reflex ipsilateral and contralateral to the electrical stimulus in mV·s (Table e.7 and Table e.8) and proportion of maximum voluntary contraction (Table e.9 and Table e.10).
