# Supplementary material for: Ultraviolet B Treatment of the Forearm Alters Supraspinal Nociceptive Processing
Source: Pain Res Manag. 2025 Jul 16;2025:6601529. doi: 10.1155/prm/6601529 (PMC12286694; doi:10.1155/prm/6601529)
Supplement: Supporting Information — Additional supporting information can be found online in the Supporting Information section. [file 6601529.f1.zip › Table e.1.docx]

Table e.1

Descriptive statistics for psychophysical assessments

|  | Mean ± standard deviation | | | | | |
| --- | --- | --- | --- | --- | --- | --- |
|  | Pinprick sharpness (0-10) | | Heat-pain (0-10) | | Pressure-pain (kg) | |
|  | Session 1 | Session 2 | Session 1 | Session 2 | Session 1 | Session 2 |
| Forearm |  |  |  |  |  |  |
| UVB-treated site | 1.35 ± 1.60 | 2.26 ± 1.90 | 1.94 ± 2.11 | 3.68 ± 2.06 | 1.62 ± 1.01 | 1.42 ± .89 |
| Ipsilateral secondary site | 1.65 ± 1.54 | 2.03 ± 1.76 | 1.48 ± 1.50 | 2.19 ± 1.80 | 1.50 ± 1.04 | 1.58 ± .96 |
| Contralateral sites | 1.42 ± 1.56 | 1.74 ± 1.75 | 1.81 ± 1.56 | 1.74 ± 1.77 | 1.57 ± 0.96 | 1.62 ± 1.09 |
| Forehead |  |  |  |  |  |  |
| Ipsilateral | 1.35 ± 1.47 | 1.48 ± 1.50 |  |  | 1.02 ± .54 | .96 ± .48 |
| Contralateral | 1.13 ± 1.34 | 1.61 ± 1.67 |  |  | 1.04 ± .62 | 1.06 ± .59 |
