# Supplementary material for: Ultraviolet B Treatment of the Forearm Alters Supraspinal Nociceptive Processing
Source: Pain Res Manag. 2025 Jul 16;2025:6601529. doi: 10.1155/prm/6601529 (PMC12286694; doi:10.1155/prm/6601529)
Supplement: Supporting Information — Additional supporting information can be found online in the Supporting Information section. [file 6601529.f1.zip › Table e.10.docx]

Table e.10

F ratios for the R2 and R3 components of the blink reflex ipsilateral and contralateral to the electrical stimulus (proportion of maximum voluntary contraction)

|  | F Ratio (1, 29 degrees of freedom) | |
| --- | --- | --- |
|  | R2 | R3 |
| Session | 6.58 * | 7.58 ** |
| Acoustic | 82.5 *** | 1.32 |
| Side | .71 | .64 |
| I/C Response | 79.8 *** | 47.3 *** |
| Session x Acoustic | 3.27 | .24 |
| Session x Side | 3.82 | 5.49 * |
| Acoustic x Side | .03 | 1.84 |
| Session x Acoustic x Side | 2.56 | 2.10 |
| Session x I/C Response | 2.76 | 3.03 |
| Acoustic x I/C Response | 10.5 ** | .03 |
| Session x Acoustic x I/C Response | .73 | .03 |
| Side x I/C Response | .63 | 1.50 |
| Session x Side x I/C Response | .51 | .69 |
| Acoustic x Side x I/C Response | .07 | 1.27 |
| Session x Acoustic x Side x I/C Response | .31 | .51 |

I/C Response: response ipsilateral versus contralateral to the electrical stimulus.

* p < .05; ** p < .01; *** p < .001
