# Supplementary material for: Ultraviolet B Treatment of the Forearm Alters Supraspinal Nociceptive Processing
Source: Pain Res Manag. 2025 Jul 16;2025:6601529. doi: 10.1155/prm/6601529 (PMC12286694; doi:10.1155/prm/6601529)
Supplement: Supporting Information — Additional supporting information can be found online in the Supporting Information section. [file 6601529.f1.zip › Table e.2.docx]

Table e.2

F ratios for psychophysical assessments

|  | F Ratio (degrees of freedom) | | |
| --- | --- | --- | --- |
|  | Session | Site | Session x Site |
| **Forearm** |  |  |  |
| Pinprick sharpness | 7.58 (1, 30), p = .010 | 3.96 (2, 60), p = .024 | 4.44 (1.53, 45.81), p = .026 |
| Heat-pain | 7.84 (1, 30), p = .009 | 12.95 (2, 60), p < .001 | 8.71 (1.62, 48.57), p = .001 |
| Pressure-pain | .50 (1, 30), p = .483 | 1.73 (2, 60), p = .186 | 3.08 (2, 60), p = .053 |
| **Forehead** |  |  |  |
| Pressure-pain | .09 (1, 30), p = .764 | 2.46 (1, 30), p = .127 | 5.63 (1, 30), p = .024 |
| Pinprick sharpness | 2.74 (1, 30), p = .108 | .12 (1, 30), p = .728 | 1.56 (1, 30), p = .221 |

* p < .05; ** p < .01; *** p < .001
