# Supplementary material for: Ultraviolet B Treatment of the Forearm Alters Supraspinal Nociceptive Processing
Source: Pain Res Manag. 2025 Jul 16;2025:6601529. doi: 10.1155/prm/6601529 (PMC12286694; doi:10.1155/prm/6601529)
Supplement: Supporting Information — Additional supporting information can be found online in the Supporting Information section. [file 6601529.f1.zip › Table e.3.docx]

Table e.3

Descriptive statistics for pain ratings to electrical stimuli ipsilateral and contralateral to the treated arm, and loudness and auditory discomfort to acoustic stimuli

|  | Mean ± standard deviation (0-10 ratings) | | | |
| --- | --- | --- | --- | --- |
|  | Session 1 | | Session 2 | |
|  | Ipsilateral stimulus | Contralateral stimulus | Ipsilateral stimulus | Contralateral stimulus |
| **Pain** |  |  |  |  |
| Electrical | 3.61 ± 1.91 | 3.43 ± 1.76 | 3.17 ± 1.68 | 3.05 ± 1.65 |
| Electrical + acoustic | 3.62 ± 1.96 | 3.42 ± 1.80 | 3.42 ± 1.80 | 3.23 ± 1.75 |
| **Loudness** |  |  |  |  |
| Acoustic | 4.78 ± 1.83 | | 4.18 ± 1.85 | |
| Electrical + acoustic | 4.70 ± 2.00 | 4.56 ± 1.92 | 4.29 ± 2.01 | 4.28 ± 2.00 |
| **Auditory discomfort** |  |  |  |  |
| Acoustic | 3.91 ± 2.36 | | 3.38 ± 2.02 | |
| Electrical + acoustic | 4.43 ± 2.30 | 4.29 ± 2.20 | 3.92 ± 2.36 | 3.97 ± 2.33 |
