# Supplementary material for: Ultraviolet B Treatment of the Forearm Alters Supraspinal Nociceptive Processing
Source: Pain Res Manag. 2025 Jul 16;2025:6601529. doi: 10.1155/prm/6601529 (PMC12286694; doi:10.1155/prm/6601529)
Supplement: Supporting Information — Additional supporting information can be found online in the Supporting Information section. [file 6601529.f1.zip › Table e.4.docx]

Table e.4

F ratios for pain ratings to electrical stimuli and loudness and auditory discomfort

to acoustic Stimulus stimuli

|  | F Ratio ^a^ | | |
| --- | --- | --- | --- |
|  | Electrical stimulus | Loudness | Discomfort |
| Session | 9.31 ** | 7.34 * | 5.46 * |
| Acoustic | 5.31 | 2.03 | 7.77 ** |
| Side | 2.64 | 5.16 * | 5.38 * |
| Session x Acoustic | 9.42 ** |  |  |
| Session x Side | .04 |  |  |
| Acoustic x Side | .49 |  |  |
| Session x Acoustic x Side | .21 |  |  |

^a^ F ratios had 1, 30 degrees of freedom except for ratings of loudness and auditory discomfort where F ratios involving the Acoustic Stimulus had 2, 29 degrees of freedom.

* p < .05; ** p < .01
