# Supplementary material for: Ultraviolet B Treatment of the Forearm Alters Supraspinal Nociceptive Processing
Source: Pain Res Manag. 2025 Jul 16;2025:6601529. doi: 10.1155/prm/6601529 (PMC12286694; doi:10.1155/prm/6601529)
Supplement: Supporting Information — Additional supporting information can be found online in the Supporting Information section. [file 6601529.f1.zip › Table e.5.docx]

Table e.5

Descriptive statistics for the R2 and R3 components of the blink reflex to the acoustic stimulus

|  | Mean ± standard deviation | | | |
| --- | --- | --- | --- | --- |
|  | Session 1 | | Session 2 | |
|  | Ipsilateral response | Contralateral response | Ipsilateral response | Contralateral response |
| **R2** |  |  |  |  |
| mV·s | 2.25 ± 1.27 | 2.54 ± 1.69 | 2.27 ± 1.03 | 2.12 ± .89 |
| Proportion of MVC | .78 ± .55 | .88 ± .66 | .62 ± .43 | .56 ± .38 |
| **R3** |  |  |  |  |
| mV·s | .68 ± .52 | .76 ± .64 | .54 ± .30 | .50 ± .22 |
| Proportion of MVC | .24 ± .24 | .25 ± .21 | .16 ± .17 | .14 ± .13 |

MVC: maximum voluntary contraction
