# Supplementary material for: Ultraviolet B Treatment of the Forearm Alters Supraspinal Nociceptive Processing
Source: Pain Res Manag. 2025 Jul 16;2025:6601529. doi: 10.1155/prm/6601529 (PMC12286694; doi:10.1155/prm/6601529)
Supplement: Supporting Information — Additional supporting information can be found online in the Supporting Information section. [file 6601529.f1.zip › Table e.6.docx]

Table e.6

F ratios for the R2 and R3 components of the blink reflex to the acoustic stimulus

|  | F Ratio (1, 29 degrees of freedom) | | | |
| --- | --- | --- | --- | --- |
|  | R2 | | R3 | |
|  | mV·s | Proportion of MVC | mV·s | Proportion of MVC |
| Session | .71 | 5.18 * | 3.93 | 3.92 |
| Side | .29 | .27 | .32 | .02 |
| Session x Side | 7.16 * | 5.34 * | 5.44 * | 1.80 |

* p < .05.
