# Supplementary material for: Ultraviolet B Treatment of the Forearm Alters Supraspinal Nociceptive Processing
Source: Pain Res Manag. 2025 Jul 16;2025:6601529. doi: 10.1155/prm/6601529 (PMC12286694; doi:10.1155/prm/6601529)
Supplement: Supporting Information — Additional supporting information can be found online in the Supporting Information section. [file 6601529.f1.zip › Table e.7.docx]

Table e.7

Descriptive statistics for the R2 and R3 components of the blink reflex ipsilateral and contralateral to the electrical stimulus (mV·s)

| Electrical stimulus ipsilateral or contralateral to the forearm treated with ultraviolet B radiation | Mean ± standard deviation (mV·s) | | | |
| --- | --- | --- | --- | --- |
|  | Session 1 | | Session 2 | |
|  | Ipsilateral response | Contralateral response | Ipsilateral response | Contralateral response |
| **R2** |  |  |  |  |
| Ipsilateral electrical stimulus | 2.52 ± 1.25 | 1.95 ± 1.17 | 2.45 ± 1.08 | 1.99 ± .89 |
| Contralateral electrical stimulus | 2.66 ± 1.58 | 2.25 ± 1.49 | 2.32 ± .84 | 1.96 ± .96 |
| Ipsilateral electrical + bilateral acoustic stimulus | 3.50 ± 1.70 | 3.08 ± 1.59 | 3.37 ± 1.45 | 3.08 ± 1.35 |
| Contralateral electrical + bilateral acoustic stimulus | 3.67 ± 1.98 | 3.37 ± 1.98 | 3.18 ± 1.25 | 2.90 ± 1.13 |
| **R3** |  |  |  |  |
| Ipsilateral electrical stimulus | 1.85 ± 1.30 | 1.50 ± 1.27 | 1.70 ± 1.31 | 1.38 ± 1.06 |
| Contralateral electrical stimulus | 2.06 ± 1.73 | 1.83 ± 1.55 | 1.54 ± 1.02 | 1.41 ± 1.08 |
| Ipsilateral electrical + bilateral acoustic stimulus | 1.76 ± 1.50 | 1.52 ± 1.42 | 1.46 ± 1.22 | 1.23 ± 1.11 |
| Contralateral electrical + bilateral acoustic stimulus | 2.02 ± 1.87 | 1.71 ± 1.72 | 1.38 ± 1.08 | 1.14 ± .95 |
