# Supplementary material for: Ultraviolet B Treatment of the Forearm Alters Supraspinal Nociceptive Processing
Source: Pain Res Manag. 2025 Jul 16;2025:6601529. doi: 10.1155/prm/6601529 (PMC12286694; doi:10.1155/prm/6601529)
Supplement: Supporting Information — Additional supporting information can be found online in the Supporting Information section. [file 6601529.f1.zip › Table e.8.docx]

Table e.8

F ratios for the R2 and R3 components of the blink reflex ipsilateral and contralateral to the electrical stimulus (mV·s)

|  | F Ratio (1, 29 degrees of freedom) | |
| --- | --- | --- |
|  | R2 | R3 |
| Session | 1.53 | 7.44 * |
| Acoustic | 109.7 *** | 1.53 |
| Side | .11 | .82 |
| I/C Response | 191.6 *** | 81.6 *** |
| Session x Acoustic | .69 | 1.48 |
| Session x Side | 2.96 | 4.90 * |
| Acoustic x Side | .35 | 1.06 |
| Session x Acoustic x Side | .92 | .21 |
| Session x I/C Response | 5.92 * | 2.55 |
| Acoustic x I/C Response | 6.55 * | .01 |
| Session x Acoustic x I/C Response | .00 | .22 |
| Side x I/C Response | 2.06 | .21 |
| Session x side x I/C Response | .47 | .12 |
| Acoustic x Side x I/C Response | .53 | 2.71 |
| Session x Acoustic x Side x I/C Response | .08 | .01 |

I/C Response: response ipsilateral versus contralateral to the electrical stimulus.

* p < .05; ** p < .01; *** p < .001
