# Supplementary material for: Ultraviolet B Treatment of the Forearm Alters Supraspinal Nociceptive Processing
Source: Pain Res Manag. 2025 Jul 16;2025:6601529. doi: 10.1155/prm/6601529 (PMC12286694; doi:10.1155/prm/6601529)
Supplement: Supporting Information — Additional supporting information can be found online in the Supporting Information section. [file 6601529.f1.zip › Table e.9.docx]

Table e.9

Descriptive statistics for the R2 and R3 components of the blink reflex ipsilateral and contralateral to the electrical stimulus (proportion of maximum voluntary contraction)

| Electrical stimulus ipsilateral or contralateral to the forearm treated with ultraviolet B radiation | Mean ± standard deviation (proportion of MVC) | | | |
| --- | --- | --- | --- | --- |
|  | Session 1 | | Session 2 | |
|  | Ipsilateral response | Contralateral response | Ipsilateral response | Contralateral response |
| **R2** |  |  |  |  |
| Ipsilateral electrical stimulus | .86 ± .55 | .67 ± .42 | .65 ± .40 | .54 ± .43 |
| Contralateral electrical stimulus | .92 ± .64 | .84 ± .78 | .62 ± .43 | .53 ± .35 |
| Ipsilateral electrical + bilateral acoustic stimulus | 1.18 ± .67 | 1.03 ± .52 | .90 ± .61 | .84 ± .67 |
| Contralateral electrical + bilateral acoustic stimulus | 1.26 ± .76 | 1.25 ± 1.04 | .84 ± .53 | .78 ± .44 |
| **R3** |  |  |  |  |
| Ipsilateral electrical stimulus | .64 ± .50 | .49 ± .35 | .47 ± .48 | .37 ± .38 |
| Contralateral electrical stimulus | .69 ± .57 | .67 ± .64 | .39 ± .30 | .38 ± .32 |
| Ipsilateral electrical + bilateral acoustic stimulus | .63 ± .60 | .48 ± .40 | .40 ± .43 | .34 ± .39 |
| Contralateral electrical + bilateral acoustic stimulus | .64 ± .51 | .62 ± .64 | .37 ± .32 | .31 ± .31 |
